# Presentation, management, and outcomes of COVID‐19 in patients with sickle cell disease

**DOI:** 10.1002/jha2.162

**Published:** 2021-01-12

**Authors:** Nwabundo Anusim, Ruby Gupta, Hyceinth Ahaneku, Candace Franklin, Savitha Balaraman, Marianne Huben, Ishmael Jaiyesimi

**Affiliations:** ^1^ Department of Hematology and Oncology Beaumont Health Royal Oak Michigan USA

**Keywords:** case series, coronavirus, COVID‐19, pandemic, Sars‐CoV‐2, sickle cell disease

The COVID‐19 virus (known as SARS‐CoV‐2) has preferential tropism for respiratory epithelium, resulting in acute respiratory syndrome similar to acute chest syndrome (ACS) seen in patients with sickle cell disease (SCD). Although subtle differences between the two are seen on imaging studies, the similarity in clinical features compels extreme caution when managing respiratory syndromes in SCD patients in the midst of the COVID‐19 pandemic. Additionally, patients with SCD are functionally immune compromised, predisposing them to infections including COVID‐19 [1]. The combination of immune‐compromise, increased cardiopulmonary risk, increased incidence of thrombosis in SCD patients, and COVID‐19 associated coagulopathy, makes SCD patients prone to increased mortality in the event of a COVID‐19 infection [2].

Here, we report demographics, clinical presentation, and outcomes of patients with background SCD and confirmed symptomatic COVID‐19 who presented to Beaumont Hospitals in Michigan, USA, between March 1, 2020 and July 1, 2020. Beaumont Hospitals is an urban not‐for‐profit hospital system with academic affiliation with the Oakland University‐William Beaumont School of Medicine. It is the largest hospital system serving the metropolitan Detroit, Michigan, USA area, with eight suburban hospital locations. The greater Detroit area served by Beaumont Hospitals has a population of over 5 million, with 79% Black race. The incidence of new diagnosis of sickle cell anemia is the highest in the State of Michigan within the city of Detroit, at 12.4 per 10,000 live births recorded in 2014. Sickle cell trait was diagnosed in Detroit in 581.4 per 10,000 births [3].

During this study period, a total of 11 patients with sickle cell disease were identified as having a positive SARS‐Cov19 polymerase chain reaction test (Table 1). All were Black race, predominantly female (7/11, 64%) with a mean (range) age of 44 (22–60) years and mean BMI of 30.2 kg/m^2^. Genotypes identified were HbSS in five (45%) patients, HbSC in four (36%), HbS/β‐thalassemia (+) in one (9%), and HbS/α‐thalassemia in one (9%). Seventy‐two percent of patients reported at least one complication of sickle cell disease and all patients had been evaluated at least once by a hematologist since the diagnosis of SCD; none of the patients were receiving chronic transfusions, hydroxyurea, voxeloter, l‐glutamine oral powder, or crizanlizumab at admission.

**TABLE 1:** Baseline characteristics and outcomes of sickle cell patients with confirmed COVID‐19

Case	Sex	Age	Genotype	BMI, kg/m^2^	Sickle cell complications	ICU care	Transfusion	Management	Length of stay (days)	Discharge Status
#1	M	48	SC	30.9	2 VOC in 10 years, splenectomy	–	–	Pain medication, zyvox, cefepime, anticoagulation, and IVF	2 admissions over 16 days	Home
#2	F	48	SS	22.6	2 VOC in 5 years	–	Exchange	Pain medication, IVF, azithromycin, hydroxychloroquine, Solu‐medrol	2 admissions over 25 days	Home
#3	F	22	SC	24.03	4 VOC in 3 years	–	–	Pain medication, IVF, azithromycin, Tamiflu, and ibuprofen	3 presentations over 7 days, no admissions	Deceased
#4	F	23	SS	40.6	ACS 10 years prior, splenectomy, multiple VOC, AVN of hip	–	–	Pain medication, anticoagulation, azithromycin, hydroxychloroquine, IVF, Solu‐medrol, ceftriaxone	10	Home
#5	F	43	SS	25.51	AVN, iron overload	–	Simple	Pain medication, IVF, azithromycin	3	Home
#6	M	54	S/alpha thalassemia	36.4	None	–	–	Pain medication, IVF, azithromycin, ceftriaxone, hydroxychloroquine	9	Home
#7	F	51	SC	26.4	AVN of hip and shoulder, multiple PE on apixaban	–	–	Pain medication, IVF, azithromycin	2	Home
#8	F	60	SS	43.35	None	Y	Exchange/ simple	Pain medication, IVF, azithromycin, ceftriaxone, hydroxychloroquine, renal dialysis	39	LTACH
#9	F	36	SS	25.31	None	–	Simple	Pain medication, IVF, azithromycin, hydroxychloroquine	5	Home
#10	M	58	S/beta (+) thalassemia	29.18	Ischemic CVA x 3 with residual left hemiparesis, chronic debility	Y	Exchange	Pain medication, IVF, azithromycin, hydroxychloroquine, Solu‐medrol	9	Deceased
#11	M	43	SC	28.45	None	–	–	Pain medication, azithromycin and hydroxychloroquine	4	Home

Abbreviations: ACS, acute chest syndrome; AVN, avascular necrosis; CT, computed tomography scan; CVA, cerebrovascular accident; CXR, chest X‐ray; IVF, intravenous fluids; LTACH, long‐term assisted care hospital; PE, pulmonary embolism; SpO2, oxygen saturation; VOC, vaso‐occlusive crisis.

The most common clinical presentations were fever, chest pain, chills, exertional shortness of breath, and cough but this was not consistent across all patients. Two patients presented with bone pain and were incidentally found to have imaging features of COVID‐19. All patients were managed with intravenous hydration and pain management as well as hydroxychloroquine (Plaquenil) 400 mg PO BID × 2, then 200 mg PO BID × 4 days, plus azithromycin 500 mg PO × 1, and then 250 mg PO daily for 4 days per institutional guidelines in place at the time of treatment. No patients were prescribed remdesivir during this time period.

Three patients (1–3) had recurrent visits to the hospital with respiratory symptoms and bone pain crises. Case 1: This patient was discharged on day 4 and was re‐admitted on day 7 with complaints of left rib pain worse on inspiration, chest tightness, and shortness of breath. Vital signs revealed a respiratory rate of 20 breaths per minute, temperature of 97.9°F, and SpO_2_ of 95% on 2 L/min supplemental oxygen. A computed tomographic (CT) scan showed a fairly extensive bilateral pulmonary emboli and multifocal basal and peripheral predominant mixed solid and ground‐glass lung opacities. He was managed with anticoagulation and discharged on Day 16 when vital signs improved and he required no supplemental oxygen.

Case 2: The patient was discharged home on day 10 when hemodynamically stable. She re‐presented on day 13 due to chest pain, back pain, and left side pain worse on deep inspiration, which were atypical of her sickle cell crises and not improving with pain medication. In the emergency department (ED), her temperature was 99.6°F, respiratory rate (RR) was 16 breaths per minute, and SpO_2_ was 99% on room air. Laboratory results revealed D‐dimer 1683 ng/dl, ferritin 1461 ng/ml, LDH 389 U/L, and electrocardiogram showed sinus rhythm. A CXR demonstrated bibasilar patchy opacities similar to the prior study. She was admitted and received ceftriaxone (Rocephin) and azithromycin for possible pneumonia. Symptoms resolved and she was discharged on day 14. On day 18, she presented with two‐ to three‐word conversational dyspnea. Physical examination revealed decreased air entry bilaterally and vital signs showed a temperature of 100.1°F, RR of 26 breaths per minute, heart rate of 116 beats per minute (bpm), and SpO_2_ of 98% on 2 L/min supplemental oxygen. Laboratory results revealed increased levels of D‐dimer of 3514 ng/dl, ferritin of 2241 ng/ml, and LDH 1341 U/L. She was continued on ceftriaxone and azithromycin during exchange blood transfusion. Hydroxychloroquine treatment (per initial institutional protocol) and methylprednisolone sodium succinate (Solu Medrol) were initiated. Symptoms significantly improved and she was hemodynamically stable and discharged on day 23. On day 25, she presented to the ED with chills, fatigue, cough, and shortness of breath with tachycardia but was otherwise stable. She elected not to be admitted and planned to follow up with her primary care physician the following day.

Case 3: A 22‐year‐old African American female with hemoglobin genotype SC presented with a 2‐week history of fever and back pain that had progressively worsened, and a 2‐ to 3‐day history of cough and increased work of breathing. Physical examination was significant for a temperature of 100.1°F, RR of 22 breaths per minute, and SpO_2_ of 97%, while a CXR showed an increased patchy bibasilar airspace disease. She was given azithromycin, oseltamivir phosphate, and ibuprofen and discharged home. On day 4, she represented with lower back pain, cough, and shortness of breath. Physical examination revealed a temperature of 98.9°F, RR of 24 breaths per minute, and SpO_2_ of 94%; breathing was unlabored and air entry was equal bilaterally. A CXR was similar to prior examination. The patient was hydrated and pain medication was administered. She was discharged home and advised to return if her SpO_2_ trend down to <92%. On day 3, she became unresponsive and was pronounced dead on arrival to the hospital.

Two patients (no. 5 and 7) presented with bone pain crisis and no respiratory symptoms, but chest imaging was suggestive of COVID‐19 infection necessitating treatment with antibiotics. In terms of thrombosis, only one patient (no. 1) was diagnosed with a pulmonary embolus on re‐admission. Five patients required transfusion (simple and/or exchange) of packed RBC of which four had HbSS genotype, and one was hemoglobin S/β‐thalassemia (+). Three patients (no. 2, 8, and 10) who were more advanced in age required exchange blood transfusion. Age was not a criterion for exchange blood transfusion; however, those patients to whom exchange blood transfusion was administered were between 48 and 60 years of age. Of those three patients, two (no. 8 and 10) required ICU admission. Patient 8 had the longest hospital stay, which was complicated by renal failure and polyneuropathy. She was discharged to a long‐term acute care facility.

Two patients (no. 3 and 10) succumbed to COVID‐19. Case 3 was described above. Case 10: A 58‐year‐old African American male, with hemoglobin genotype S/β‐thalassemia (+) and a history of three cerebrovascular accidents with residual left‐sided hemiplegia presented to the ED from a long‐term acute care hospital with altered mental status, fever, and acute hypoxic respiratory failure requiring emergent intubation. Laboratory values were significant for an elevated D‐dimer of >10,000 ng/dl, ferritin of 9681 ng/ml, LDH of 784 U/L, and hemoglobin of 8.1 g/dl. A CXR showed bilateral airspace opacifications with SARS‐CoV‐2 PCR test positive on admission. The patient was admitted to the intensive care unit (ICU) for acute respiratory failure where he was treated with azithromycin, hydroxychloroquine, and methylprednisolone sodium succinate until clinical status deteriorated and he unfortunately expired after a 9‐day hospital course.

None of our patients were on sickle cell disease modifying agents, hence data are insufficient to prove whether prophylactic SCD medications reduce the morbidity from COVID‐19 among SCD patients. Our study outcome shows that patient presentations varied with some patients having no respiratory symptoms while others have severe symptoms requiring care in the intensive care unit. Paradoxically, the two patients who succumbed to the infection had milder sickle cell disease (HgSC and Hg S/β‐thalassemia (+)). The history of prior sickle cell complications such as avascular necrosis of the joints, hypersplenism requiring splenectomy, cerebrovascular accident, and the sickle cell genotype did not determine the outcome of patients with COVID‐19. It is also interesting to note that patients who presented with bone pain crises and no respiratory symptoms tested positive for COVID‐19 and imaging revealed findings similar to COVID‐19 pulmonary infection. Lengthier hospital stays were noted in patients of older age compared to their younger counterparts (Table 1), which is consistent with current data showing that the elderly and unfit patients are more likely to have a higher morbidity and mortality with COVID‐19 [4].

Almost half the patients received blood transfusion as part of their management. The threshold for simple transfusion was haemoglobin of 7 g/dl and exchange transfusion was offered to two patients with decline in cardiopulmonary status. Hence, it is important to differentiate ACS from COVID‐19 pneumonia and institute early exchange or simple blood transfusion in addition to respiratory interventions, thromboprophylaxis, and continue sickle cell disease‐modifying agents if the patient was already receiving those medications [5]. In a comprehensive multicentre review of 19 sickle cell patients with COVID‐19, each center treated patients rather differently without a guiding protocol [4]. The American Society of Hematology provides resources for the management of SCD patients with COVID‐19 [6]. We continue to seek to improve management of sickle cell patients and we encourage patients to participate in clinical trials.
